# Clinical Use of Nomogram Based on Machine Learning for Diagnosis Prediction of Acute Respiratory Distress Syndrome in Patients With Acute Pancreatitis

**DOI:** 10.1155/mi/5610316

**Published:** 2025-11-17

**Authors:** Hongjie Hu, Yuxin Wang, Yaqin Song, Shuhui Wu, Dayong Li, Liang Jing, Lei Qin, Zhaohui Xia, Wei Zhu

**Affiliations:** ^1^Department of Emergency Medicine, Tongji Hospital, Tongji Medical College, Huazhong University of Science and Technology, Wuhan, Hubei, China; ^2^Department of Intensive Care Medicine, Tongji Hospital, Tongji Medical College, Huazhong University of Science and Technology, Wuhan, Hubei, China; ^3^School of Biomedical Engineering, Guangzhou Medical University, Guangzhou, Guangdong, China; ^4^School of Mechanical Science and Engineering, Huazhong University of Science and Technology, Wuhan, Hubei, China

**Keywords:** acute pancreatitis, acute respiratory distress syndrome, artificial intelligence, machine learning, nomogram

## Abstract

**Background:**

This study focused on utilizing machine learning techniques to construct a predictive nomograph for early identification of acute pancreatitis (AP) patients at risk of developing acute respiratory distress syndrome (ARDS).

**Methods:**

We retrospectively analyzed 427 AP patients from Tongji Hospital (2010–2021) and externally validated the model using the MIMIC-IV database. Six machine learning algorithms were compared, with the support vector machine (SVM) selected for nomogram construction. Key predictors included age, sex, SOFA score, C-reactive protein (CRP), platelet count (PLT), total bilirubin (TBIL), and direct bilirubin (DBIL). Model performance was assessed via area under the curve (AUC), calibration curves, and decision curve analysis (DCA).

**Results:**

The SVM model exhibited the best performance among six machine-learning models assessed. Key predictors including age, sex, SOFA, CRP, PLT, TBIL, and DBIL levels were incorporated into the nomogram. The nomogram demonstrated good discriminatory ability and clinical applicability, with a C-index of 0.818 in the training cohort and 0.799 in the testing cohort. External validation using the MIMIC IV database further confirmed its accuracy, with a C-index of 0.759. Notably, calibration curves showed excellent agreement between predicted and observed outcomes, and DCA indicated a favorable net benefit, reinforcing the model's reliability.

**Conclusions:**

The prediction nomogram constructed based on the SVM model in this study can effectively predict the probability of AP complicated by ARDS.

## 1. Introduction

Acute pancreatitis (AP) is a prevalent critical disease. Globally, approximately 10%–20% of patients with AP experience a complex multiple organ dysfunction syndrome (MODS), with a mortality rate of 10%–15%, even reaching as high as 36%–50% [1, 2]. Acute respiratory distress syndrome (ARDS) is a life-threatening clinical syndrome, and the in-hospital mortality rate can reach 35%–45% [3, 4]. Respiratory failure is the most frequent organ dysfunction in the early and late stages of AP, with high mortality [5, 6]. A previous study has indicated that up to 60% of deaths within the first week of AP are considered to be caused by acute lung injury (ALI) associated with pancreatitis and ARDS [7]. In addition, a series of studies found that early intervention of ARDS can effectively improve the prognosis of patients with AP [8, 9]. Therefore, early warning of the risk of ARDS in patients with AP and timely intervention can effectively reduce the incidence of concurrent ARDS in AP and improve prognosis.

Nomograms, as a statistical model based on various clinical variables, have been designed to predict the complications, prognosis, and survival rates in numerous diseases [10–12]. The nomogram approach has shown good prediction and prognostic evaluation functions for cancer, sepsis, and other diseases. In recent years, machine learning methods have acquired increasing popularity in the field of medical research [13, 14]. Compared to traditional statistical approaches, machine learning methods offer higher precision in predicting patient outcomes, as well as greater accuracy and adaptability, even with a limited number of input factors. As a result, more and more researchers are applying these techniques in clinical settings, particularly in intensive care medicine [15–17]. Nomograms and machine learning methods can reasonably predict the onset and progression of disease. However, to date, no study has combined nomogram development with machine learning to predict the early occurrence of ARDS in patients with AP.

ARDS is the most common type of organ failure in patients with AP and remains the major reason for high in-hospital mortality to this day [8]. The treatment of ARDS typically requires a significant amount of medical resources, including intensive care unit (ICU) beds, ventilators, the time of doctors and nurses, and so on, which increases healthcare costs. An increasing number of studies have identified factors such as diffuse alveolar damage, bacteremia, and intestinal barrier dysfunction as potential causes of AP-related ARDS, but the specific mechanisms remain unclear [18]. Therefore, the treatment of ARDS caused by AP is extremely challenging, and early recognition of ARDS is also essential. The use of predictive models for AP-related ARDS will help in the early identification and classification of high-risk patients, enabling early intervention measures such as mechanical ventilation, oxygen therapy, and thus improving the chances of survival for patients. ARDS prediction models can be utilized for investigating the pathogenesis, treatment methods, and prognostic factors of ARDS. Previous researcher has attempted to predict the risk of developing ARDS in AP using clinical imaging examinations features [19]. Up to now, no scholars have utilized machine learning models in conjunction with clinical features to predict ARDS in AP. In this study, we used machine learning to analyze and classify variables involving clinical and laboratory characteristics of patients with AP with ARDS to build a nomogram able to predict the risk of developing ARDS in these patients. The main purpose of this study was to allow the timely detection of ARDS and to improve the medical prognosis of patients with AP through early intervention.

## 2. Methods

### 2.1. Patient Selection and Study Design

This study was designed as a retrospective investigation of data from patients enrolled with AP treated at Tongji Hospital, Tongji Medical College, Huazhong University of Science and Technology. The study was conducted according to the Declaration of Helsinki. The study protocol was approved by the Ethics Committee of Tongji Hospital (TJ-IRB20220102) with a waiver for informed consent. We followed the TRIPOD guidelines for reporting (Supporting Information 1: Tripod Checklist).

**Figure 1:**
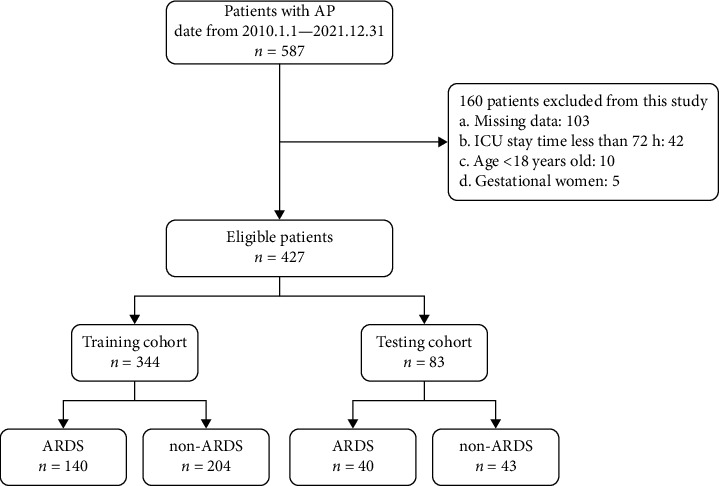
Flowchart for modeling set patient selection.

**Figure 2:**
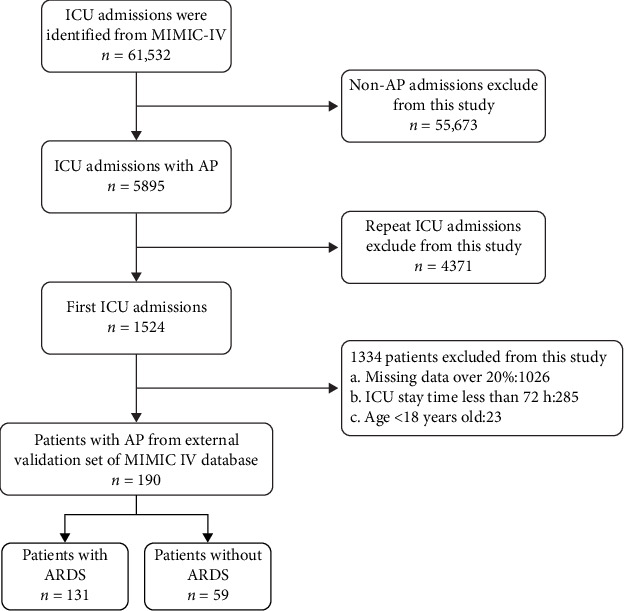
Flowchart for external validation set patient selection.

During the model training phase, a total of 587 patients were registered in this study, and 427 of these patients were included in the final analysis. We divided the dataset into two main subsets, with the first subset comprising the training and validation sets, and the second subset including the test set. In the first subset used for model training, a total of 344 patients were assigned to the training and validation cohorts, while 83 patients were allocated to the testing cohort. In the first subset, the patients were hospitalized at Tongji Hospital, Tongji Medical College, Huazhong University of Science and Technology, China, between 1 January 2010 and 28 February 2021. Data from the first subset were split into 80% as the training set and 20% as the validation cohort. In the internal testing cohort, the patients were hospitalized at Tongji Hospital, Tongji Medical College, Huazhong University of Science and Technology, China, between 1 March 2021 and 31 December 2021. The inclusive criteria were: (1) diagnosed as AP at admission, but not as ARDS, (2) age > 18 years, (3) ICU stay > 72 h, and (4) patients entered the ICU within less than 24 h following diagnosis. The exclusion criteria were as follows: (1) chronic pancreatitis or an acute attack of chronic pancreatitis, (2) patients combined with advanced tumors, (3) gestational women, and (4) missing clinical data. Furthermore, a total of 190 patients from the MIMIC IV database were included in the study as the external testing cohort. Figures 1 and 2 show the flowchart used for patient selection.

### 2.2. Data Collection

The selection of potential clinical variables was informed by existing research findings, practical accessibility, and expert domain knowledge, and we collected 31 potential characteristics according to the above requirements [20]. All these characteristics were derived from the dataset of patients in the hospital's electronic medical record system within the first day of admission. For the selected variables, variance inflation factor (VIF) was used to detect collinearity, and the results indicated that the VIFs of the variables were all below 10 and no collinearity existed (Supporting Information 2: Table S1). Demographic characteristics included sex, age, the Glasgow coma scale (GCS), the Acute Physiology and Chronic Health Evaluation II (APACHE II) score, and the sequential organ failure score (SOFA). Vital signs included heart rate (HR), respiratory rate (RR), body temperature (T), systolic blood pressure (SBP), diastolic blood pressure (DBP), and mean arterial pressure (MAP). Laboratory tests within 24 h after admission included platelet count (PLT), red blood cell count (RBC), white blood cell count (WBC), hemoglobin (Hb), alanine transaminase (ALT), aspartate transaminase (AST), total bilirubin (TBIL), direct bilirubin (DBIL), blood urea nitrogen (BUN), serum creatinine (Cr), C-reactive protein (CRP), B-type natriuretic peptide (BNP), procalcitonin (PCT), serum amylase (AMY), serum lipase (LIP), blood coagulation factor and D-dimer. To improve model training and reduce the potential bias of overfitting data, the data was normalized to a scale of (0, 1) for each characteristic.

During the data processing stage, we utilized the Python programing language to impute missing values. Missing data percentages per variable are shown in Supporting Information 3: Figure S1. For variables with missing values not exceeding 20%, we opted for the multiple imputation method to ensure data integrity and reliability [21, 22]. When dealing with variables that have multiple measurements, we have opted to include the extreme values from the various measurements. In cases where only a single measurement is available, we have chosen to incorporate that specific measurement result. However, for indicators with significant amounts of missing data, such as TG, we chose not to perform data imputation, as this could potentially introduce certain biases to the results.

### 2.3. Diagnosis of AP and ARDS

The diagnosis of AP was based on the revised Atlanta classification [23]. AP was diagnosed if at least two of the following three criteria were met:

1. Abdominal pain (persistent and severe epigastric pain in an acute attack, usually radiating to the back);
2. Serum lipase (or amylase) activity at least three times higher than the upper normal limit;
3. The characteristics of AP on contrast-enhanced computed tomography (CT) or magnetic resonance imaging (MRI) or transabdominal ultrasound.

According to the Berlin definition [3], the diagnostic criteria for ARDS are as follows:

1. The appearance within 1 week of a known injury or new or worsening respiratory symptoms;
2. Profound hypoxemia;
3. Bilateral lung opacities on radiological examination;
4. Respiratory failure that cannot be explained by heart failure or fluid overload.

The diagnosis time of ARDS in patients occurs from 30 to 78 h after admission.

### 2.4. Algorithm Development and Testing Procedures

For data preprocessing, each specific index of the records was converted into numerical or categorical variables using Python (version 2.8.1). Initially, LASSO regression was applied to perform feature selection, and five-fold cross-validation was used to evaluate model performance. Variables with the minimum mean square error (MSE) in the log(*λ*) were selected to build the prediction model (Supporting Information 3: Figure S2). After feature selection, six machine learning models—logistic regression, backpropagation neural network (BPNN), random forest, support vector machine (SVM), decision trees, and extreme gradient boosting network (XGBoost)—were applied to assess the model's performance based on sensitivity, specificity, F1 score, recall, precision, positive predictive value, and negative predictive value. The final candidate model was selected based on accuracy and the area under the curve (AUC). To evaluate the model's predictive ability, decision curve analysis (DCA) and calibration curves were used. SHAP (Shapley additive explanations) values were applied to analyze feature importance in the model.

### 2.5. Nomogram Construction and Validation

To provide a simple graphical representation of the prediction model, we developed a nomogram. The construction of the nomogram and the validation analysis were performed using the R statistical package v.4.2.0. The nomogram was validated using discrimination and calibration with the validation cohort. The areas under the receiver-operator characteristic (ROC) curves were calculated to evaluate the performance of the constructed nomogram. Calibration curves were plotted to visualize the agreement between predicted probabilities and observed outcomes. The Brier score (0.21 for training, 0.18 for validation) was computed to numerically assess calibration precision, with lower values indicating superior reliability. The clinical net benefit and predictive effect of the nomogram were evaluated by DCA. The goodness of fit of the model was verified by the Hosmer–Lemeshow test.

### 2.6. Model Explainability

To further interpret the machine learning model and understand the contribution of each feature, we utilized SHAP values. SHAP values provide a unified measure of feature importance by attributing the prediction of each observation to individual features, offering both global and local interpretability. In our study, SHAP analysis was applied to the final prediction model to assess how each variable influenced the model's output.

### 2.7. Statistical Analysis

The frequency and percentage of descriptive statistics were analyzed for categorical variables and evaluated using the chi-square test or Fisher's exact test. For continuous variables, means ± standard deviations and ranges, or medians and ranges, were utilized for description. When comparing continuous variables with a normal distribution, the Students' test was used; otherwise, the Mann–Whitney *U* test was employed. *p*-values < 0.05 were considered statistically significant.

## 3. Results

### 3.1. Baseline Characteristics of the Patients

Stratifying patients with AP according to ARDS presentation, 344 patients in the training cohort were classified as an ARDS group (*n* = 140) and a non-ARDS group (*n* = 204). Comparisons of demographic and clinical characteristics were made between patients with ARDS and patients without ARDS (non-ARDS) and are shown in Tables 1 and 2. In the univariate analysis, significant differences were observed for 14 of 29 potential risk factors and included characteristics, such as sex, age, APACHE II score, SOFA score, RR, CRP, PCT, WBC, PLT, ALT, AST, TBIL, and DBIL.

### 3.2. Modeling and Feature Selection

This study employed a multistage feature optimization strategy to develop and evaluate prediction models for ARDS. In the initial phase, LASSO regression with five-fold cross-validation was implemented for feature selection, the minimum mean squared error occurred at Log(*λ*) = −3.2. This process identified nine clinically relevant predictors: age, sex, SOFA score, GCS score, CRP, PLT, ALT, TBIL, and DBIL. Subsequently, six machine learning algorithms—logistic regression, BPNN, RF, SVM, decision tree, and XGBoost—were systematically evaluated for their predictive performance using these nine variables. Cross-validation results demonstrated that the SVM model achieved superior discriminative ability, with an accuracy of 0.942, precision of 0.951, and F1-score of 0.931 (Table 3), outperforming other comparative models. Given the SVM's exceptional performance, recursive feature elimination was conducted to refine the optimal feature subset. Through grid search optimization with five-fold cross-validation, a parsimonious seven-variable subset—comprising age, sex, SOFA score, CRP, PLT, TBIL, and DBIL—was identified. Multicollinearity analysis confirmed that these predictors exhibited acceptable VIFs, indicating no significant collinearity among the selected features (Supporting Information 2: Table S1). This optimized subset enhanced prediction accuracy to 0.9636 (Table 4). Finally, a clinically interpretable nomogram prediction model was constructed based on this refined feature combination, effectively balancing predictive power with model simplicity.

**Figure 3:**
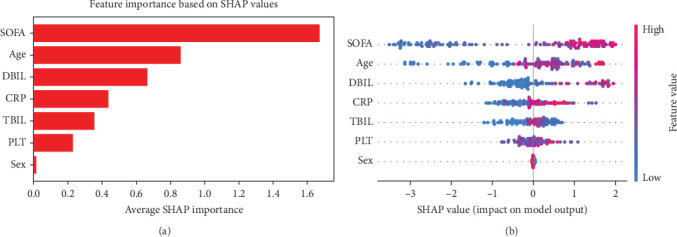
Rank the important features and the global influence of single variable in SVM model. (a) Features important ranking in SVM model; (b) positive and negative effects of variables on global outcome in SVM model.

Through the SHAP summary plot for the SVM model (Figure 3A), we identified that SOFA, age, and DBIL are the most important predictors. The SHAP summary plot highlights the average impact of these features on the model's output, indicating its critical role in the prediction process. Additionally, we used SHAP dependence plots (Figure 3B) to further illustrate how each feature globally influences the model's final predictions. These dependence plots visualize the relationship between feature values and SHAP values, providing deeper insights into how each feature affects the model's predictions across its value range.

### 3.3. Construction and Performance of the Nomogram

**Figure 4:**
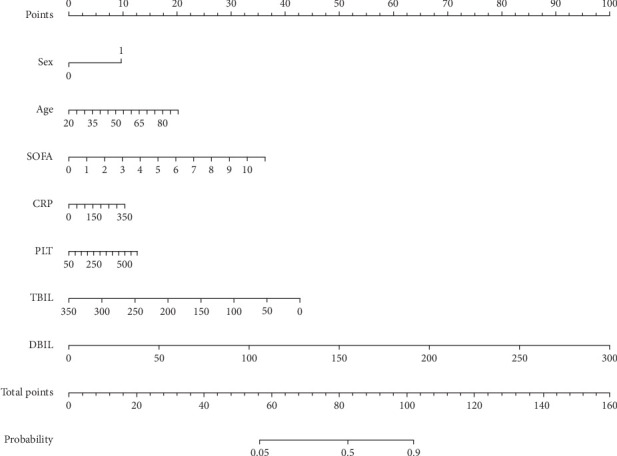
A nomogram for prediction AP patients with ARDS.

**Figure 5:**
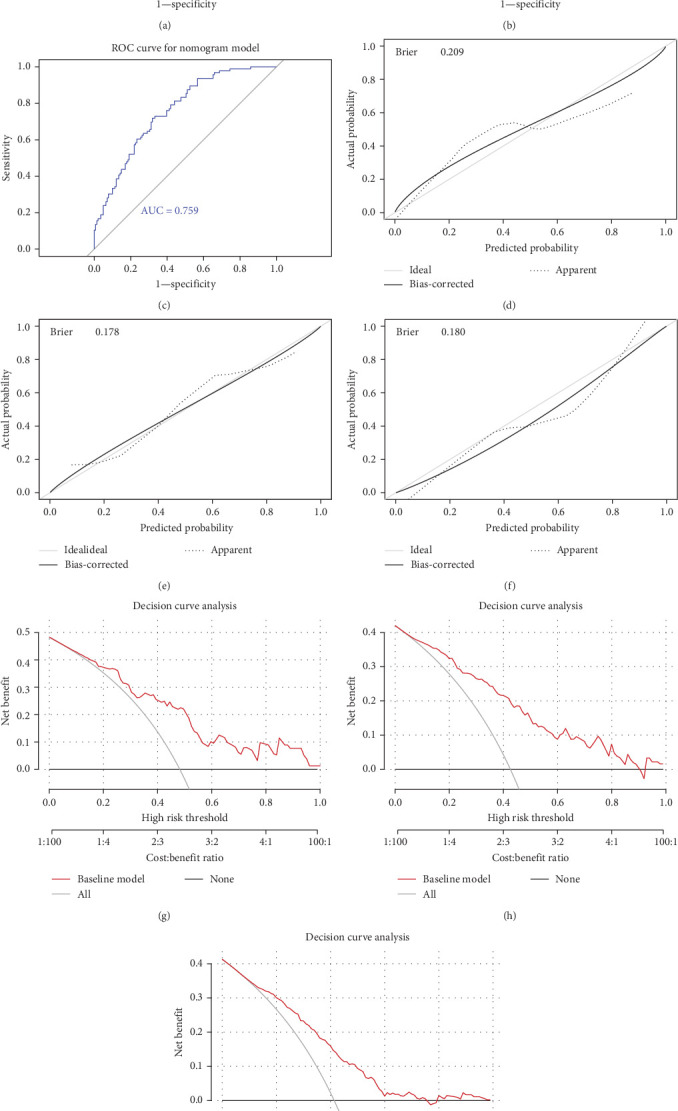
The evaluation of modeling and validation sets. The ROC curve in the training cohort (a), testing cohort (b) and external validation cohort (c). The calibration curve of the nomogram predicting AP patients with ARDS in in the training cohort (d), testing cohort (e) and external validation cohort (f). The DCA curve of the training cohort (g), testing cohort (h) and external validation cohort (i). *Y*-axis represents the net benefit.

The nomogram, depicted in Figure 4, was developed based on the seven identified characteristics. To comprehensively evaluate its predictive performance and clinical utility, we employed ROC curves, calibration curves, and DCA. The area under the ROC curve (AUC) was used as a measure of diagnostic performance, with the model achieving a C-index of 0.818 (95% CI: 0.736–0.913) in the training cohort and 0.799 (95% CI: 0.764–0.862) in the testing cohort, demonstrating strong discriminative ability. Furthermore, the calibration curves indicated excellent agreement between predicted and observed probabilities, confirming the reliability of the model. The Brier score, which quantifies the model's accuracy, remained consistently below 0.25, suggesting good overall calibration and predictive performance. During the validation process utilizing patients from the MIMIC IV database, the model demonstrated a C-index of 0.759 (95% CI: 0.708–0.842). The DCA further confirmed the consistency between the training and testing cohorts, indicating a robust performance of the model. Notably, the model exhibited excellent effectiveness in the MIMIC IV validation set, as illustrated in Figure 5, providing additional evidence of its reliability and performance.

## 4. Discussion

This study was the first to apply a machine learning-based nomogram to predict the risk of ARDS in patients with AP. The accuracy of the model shows its potential as a tool to predict the occurrence of ARDS in patients with AP. The findings demonstrated that age, sex, SOFA score, CRP, PLT, TBIL, and DBIL were predictors of whether ARDS occurs in patients with AP. Furthermore, a nomogram was constructed based on the above predictors, which facilitated individualized prediction of ARDS in patients with AP. The consequences of internal and external approvals showed that this nomogram had good predictive performance.

In our study, patients with AP complicated by ARDS were generally older than those without ARDS. Previous studies have reported that advanced age is an important risk factor for the occurrence of ARDS [24, 25]. This may be related to the fact that older patients are more likely to have underlying cardiovascular disease, less robust immune responses, poorer organ reserve function, and therefore exhibit an increased risk of occurrence of various concomitant diseases [3, 26]. When pancreatitis occurs, it is more likely to cause organ dysfunction in older patients due to other accompanying diseases, increasing the difficulty of treatment. Previous research has demonstrated the effect of sex differences on ARDS [27]. A recent study revealed that men more frequently develop ARDS when diagnosed with coronavirus disease 2019 (COVID-19) compared to women [28]; our study showed that men were more likely to develop ARDS in combination with AP. For those patients with AP admitted to the ICU, clinicians should pay closer attention to age and sex and be alert to the occurrence of ARDS.

The SOFA score was an independent predictor of the appearance of ARDS in patients with AP and carried a high weight. The SOFA score is a good tool for evaluating the condition of patients in the ICU and has good predictive capabilities for predicting severity and mortality [29–31]. Compared to the APACHE II score, the SOFA score can better predict the mortality and severity of patients in the ICU with AP [32]. Furthermore, the SOFA score also shows a good predictive effect on predicting the prognosis of ARDS [33]. Altogether these studies indicate that the SOFA score plays an important role in the prediction of AP and ARDS, which is consistent with our model.

CRP is a widely used serum biomarker for the diagnosis of infection and inflammation [34]. As an acute phase protein, the CRP level indicates the rate of release of inflammatory cells and pro-inflammatory factors, and the levels of CRP often indicate levels of systemic inflammation. Li et al. [35] showed that CRP is an independent influencing factor in predicting the occurrence of ARDS in patients with sepsis. Animal experiments showed that when AP occurs, inflammatory cells aggregate, activate, and promote the release of inflammatory cytokines [36]. The accumulation and release of these inflammatory cells and pro-inflammatory cytokines can exacerbate organ damage. CRP, as a serum biomarker of inflammation, will also increase significantly in this context, which may explain why CRP was an important predictor of whether ARDS occurs in patients with AP in our study.

There is increasing evidence for the role of platelets in ARDS morbidity and mortality [37]. In the pathogenesis of ARDS, dysregulation of coagulation and inflammatory response are critical mechanisms [38]. A study found that platelet activation and platelet-dependent monocyte tissue factor expression were closely associated with the severity and mortality of patients with COVID-19 [39]. In our study, the number of platelets in AP patients with ARDS decreased significantly, and thrombocytopenia was found to be a substantial independent risk factor for patients with AP and ARDS, suggesting that platelet activation and reduction may be involved in the pathophysiological process. Thrombocytopenia (PLT) as an independent predictor may influence ARDS progression through dual mechanisms: impaired endothelial repair due to platelet deficiency exacerbates alveolar-capillary leakage, while platelet-derived mediators such as serotonin and PF4 directly enhance pulmonary vasoconstriction and neutrophil extracellular trap (NET) formation [40]. These findings provide a rationale for exploring antiplatelet therapies in ARDS prevention.

Elevated TBIL and DBIL levels reflect cholestatic hepatic dysfunction commonly seen in severe AP, where pro-inflammatory mediators impair hepatocellular metabolism and biliary excretion pathways [41, 42]. This hepatic impairment often parallels the severity of systemic inflammatory response syndrome (SIRS), a critical driver of multiorgan dysfunction, including ARDS [43, 44]. Moreover, bilirubin metabolism serves as a sensitive indicator of oxidative stress and antioxidant capacity. Unconjugated bilirubin acts as an endogenous antioxidant, scavenging reactive oxygen species (ROS) during systemic inflammation; however, persistently elevated bilirubin signifies that hepatic clearance and antioxidant defenses are overwhelmed, thereby exacerbating oxidative injury and promoting hepatocellular damage [45, 46]. Clinical studies correlate higher bilirubin levels with pulmonary endothelial dysfunction, increased microvascular permeability, and more severe ARDS pathology in critically ill patients [47]. Elevated TBIL and DBIL have also been identified as independent prognostic markers for morbidity and mortality in MODS, with a particularly strong association with pulmonary outcomes [48]. By capturing these early pathophysiological changes—hepatic cholestasis, oxidative stress overload, and endothelial injury—TBIL and DBIL enhance our nomogram's ability to stratify ARDS risk at a stage when timely intervention can improve patient outcomes.

Some scholars have also attempted to explore the relationship between AP and the development of ARDS [49–51]. Compared to these studies, our research has a larger sample size, and encompass six different machine learning models. Moreover, we conducted both internal validation within our center's dataset and external validation using the MIMIC-IV critical care database to confirm the accuracy of our models. Furthermore, the patient cohort in our study exclusively received treatment within the ICU, with no inter-departmental transfers, which enhances the accuracy in reflecting the disease status of critically ill patients. All these aforementioned distinctions contribute to the novelty and importance of our research findings.

This study has limitations: (1) This study was conducted at a single center using retrospective data, which may introduce selection bias. The patient cohort might not fully represent other populations with different healthcare settings. Future studies should incorporate multicenter validation to improve the generalizability of the model. (2) The dataset spans from 2010 to 2021, and some ARDS cases might have been diagnosed before the Berlin definition (2012) was established. This inconsistency in diagnostic criteria may have influenced the model's performance. Future studies should use datasets based on the latest diagnostic criteria to ensure consistency. (3) Although we selected seven variables that demonstrated strong predictive power, additional biomarkers—such as Ang-2, and inflammatory cytokines (IL-6 and TNF-α)—might further enhance the model's predictive performance. Future studies should incorporate multiomics data to refine prediction accuracy. (4) This study utilized only clinical variables obtained within the first 24 h after admission. However, the dynamic changes in SOFA scores, CRP levels, and other markers over time might provide more valuable predictive insights. Future studies should explore longitudinal models or real-time risk assessment tools to capture disease progression more effectively.

The study did not perform subgroup analyses by detailed disease timing or severity due to limitations of the retrospective dataset. However, as described in the Methods, ARDS events predominantly occurred 30–78 h after ICU admission, which to some extent defines the disease stage at prediction. Importantly, first-day ICU data are critically important for disease evaluation and the allocation of limited critical-care resources. Future prospective studies with rigorous documentation of symptom onset and severity scoring are warranted to validate our model across different AP phases.

In a previous study, we reported initial findings on the use of a nomogram for diagnosing ARDS in patients with AP, which were made publicly available as a preprint [52]. The present study expands upon these findings by describe what new or additional aspects are covered in this manuscript.

## 5. Conclusion

In conclusion, this study developed a machine learning-based nomogram to predict the risk of ARDS in AP patients. The model demonstrated good predictive accuracy and clinical applicability. However, further multicenter prospective validation, incorporation of additional biomarkers, and real-world clinical testing are needed to enhance its robustness and translational impact.

## Figures and Tables

**Table 1:** Comparisons between training and testing cohorts.

Variable	Train cohort	Test cohort	*p*
Number (sample size)	344	83	—
Baseline characteristics			
Age (year, mean SD)	51 (40–65)	50 (36–62)	0.223
Sex (%)			
Female	202	37	—
Male	142	46	0.027
Etiology			
Hypertriglyceridemia	39 (11.33)	13 (15.67)	<0.001
Alcohol	75 (21.80)	27 (32.53)	—
Gallstone	110 (31.98)	8 (9.63)	—
Mixed type	34 (10.00)	15 (18.07)	—
Uncertainty	86 (25.00)	20 (24.10)	—
APACHE II score, median (IQR)	11 (6–17)	10 (5–14)	0.117
SOFA score, median (IQR)	5 (3–7)	4 (3–6)	0.073
GCS score, median (IQR)	15 (13–15)	15 (13–15)	0.072
Vital signs			
Temperature (°C), median (IQR)	36.5 (36.4–37.2)	36.8 (36.5–37.5)	0.144
Heart rate (times/min), median (IQR)	94 (80–114)	101 (89–122)	0.050
Respiratory rate (times/min), median (IQR)	20 (20–24)	20 (20–25)	0.741
SBP (mmHg), median (IQR)	126.0 (112.0–143.0)	125 (112–140)	0.839
DBP (mmHg), median (IQR)	78.0 (68.0–87.0)	75.0 (66.0–86.0)	0.282
MAP (mmHg), median (IQR)	93.5 (84.0–104.0)	91.6 (74.0–104.0)	0.621
Laboratory parameters			
D dimer (mg/L), median (IQR)	2.5 (1.3–6.0)	3.1 (1.6–6.7)	0.067
PT (s), median (IQR)	14.1 (13.6–16.9)	14.7 (13.7–15.9)	0.692
INR, median (IQR)	1.1 (1.0–1.3)	1.7 (1.0–1.2)	0.342
APTT (s), median (IQR)	41.8 (36.2–58.6)	42.2 (36.7–48.1)	0.387
TT (s), median (IQR)	16.4 (15.1–19.2)	16.4 (15.0–18.4)	0.761
BNP (pg/mL), median (IQR)	343.5 (93.5–1082.0)	332.0 (98.0–654.0)	0.471
PCT (ng/mL), median (IQR)	1.0 (0.3–5.3)	1.1 (0.3–2.8)	0.438
CRP (mg/L), median (IQR)	71.2 (25.3–177.8)	172.8 (58.4–242.9)	<0.001
AMY (U/L), median (IQR)	293.0 (86.0–921.8)	245.0 (56.0–775.0)	0.070
LIP (U/L), median (IQR)	475.2 (146.2–1663.0)	402.9 (102.5–974.7)	0.074
WBC (10^9^/L), median (IQR)	12.9 (9.5–16.8)	12.7 (8.8–15.8)	0.336
RBC (10^9^/L), median (IQR)	4.1 (3.5–4.8)	4.1 (3.6–4.7)	0.927
Hb (g/L), median (IQR)	127.0 (107.3–150.0)	129 (112–142)	0.739
PLT (10^9^/L), median (IQR)	166.5 (111.8–225.3)	171.0 (132.0–234.0)	0.131
ALT (U/L), median (IQR)	29.0 (14.0–84.7)	21.0 (12.0–55.0)	0.177
AST (U/L), median (IQR)	36.5 (21.0–102.8)	30.0 (16.0–61.0)	0.005
TBIL (μmol/L), median (IQR)	15.1 (9.8–27.6)	16.6 (9.9–28.8)	0.791
DBIL (μmol/L), median (IQR)	7.0 (3.4–16.5)	8.3 (4.2–17.1)	0.231
BUN (mmol/L), median (IQR)	7.6 (4.5–14.0)	6.8 (4.3–11.6)	0.176
Cr (μmol/L), median (IQR)	91.6 (62.2–223.5)	73.9 (56.2–157.0)	0.034

*Note:* AMY, serum amylase; Cr, serum creatinine; LIP, serum lipase.

Abbreviations: ALT, alanine transaminase; APACHE II, acute physiology and chronic health evaluation II; APTT, activated partial thromboplastin time; AST, aspartate transaminase; BNP, B-type natriuretic peptide; BUN, blood urea nitrogen; CRP, C-reactive protein; DBIL, direct bilirubin; DBP, diastolic blood pressure; GCS, Glasgow coma scale; Hb, hemoglobin; HR, heart rate; INR, International Normalized Ratio; MAP, mean arterial pressure; PCT, procalcitonin; PLT, platelet; PT, prothrombin time; RBC, red blood cell; RR, respiratory rate; SBP, systolic blood pressure; SOFA, sequential organ failure score; TBIL, total bilirubin; TT, thrombin time; WBC, white blood cell.

**Table 2:** Comparison between patients with and without ARDS in the training cohort.

Variable	ARDS	Non-ARDS	*p*
Number (sample size)	140	204	—
Baseline characteristics			
Age (year, mean SD)	52 (41–66)	50 (39–65)	0.011
Sex(%)			
Female	73	148	—
Male	67	56	<0.001
Etiology			
Hypertriglyceridemia	12 (8.50)	23 (11.27)	0.074
Alcohol	43 (30.70)	79 (38.73)	—
Gallstone	35 (25.00)	27 (13.24)	—
Mixed type	28 (20.00)	41 (20.10)	—
Uncertainty	22 (17.85)	34 (16.67)	—
APACHE II score, median (IQR)	11 (7–18)	10 (5–16)	<0.001
SOFA score, median (IQR)	5 (4–9)	4 (1–7)	<0.001
GCS score, median (IQR)	15 (13–15)	15 (13–15)	0.045
Vital signs			
Temperature (°C), median (IQR)	36.6 (36.4–37.2)	36.5 (36.5–37.3)	0.280
Heart rate (times/min), median (IQR)	98 (83–113)	97 (80–114)	0.578
Respiratory rate (times/min), median (IQR)	20 (20–25)	20 (20–23)	<0.001
SBP (mmHg), median (IQR)	125.0 (110.0–144.0)	78.0 (69.0–85.0)	0.417
DBP (mmHg), median (IQR)	77.0 (68.0–87.0)	75.0 (66.0–86.0)	0.600
MAP (mmHg), median (IQR)	90.5 (82.0–107.0)	93.8 (84.7–103.7)	0.487
Laboratory parameters			
D dimer (mg/L), median (IQR)	3.2 (1.5–7.5)	2.3 (1.0–5.2)	0.081
PT(s), median (IQR)	15.1 (14.0–17.3)	14.9 (13.6–16.5)	0.761
INR, median (IQR)	1.2 (1.1–1.4)	1.2 (1.0–1.3)	0.263
APTT(s), median (IQR)	41.1 (36.3–55.3)	41.7 (36.1–57.2)	0.213
TT(s), median (IQR)	16.9 (15.3–19.7)	16.1 (15.0–19.0)	0.479
BNP (pg/mL), median (IQR)	412.5 (108.3–1345.0)	242.5 (88.3–959.8)	0.636
PCT (ng/mL), median (IQR)	1.6 (0.3–7.7)	0.89 (0.2–4.5)	<0.001
CRP (mg/L), median (IQR)	112.8 (35.6–213.2)	61.7 (19.3–153.5)	0.028
AMY (U/L), median (IQR)	435.0 (97.3–956.5)	254.0 (80.5–897.0)	0.109
LIP (U/L), median (IQR)	458.6 (128.5–1447.0)	422.0 (92.5–897.0)	0.058
WBC (10^9^/L), median (IQR)	14.5 (10.0–18.1)	12.5 (9.1–16.2)	<0.001
RBC (10^9^/L), median (IQR)	4.1 (3.4–4.8)	4.2 (3.5–5.0)	0.180
Hb (g/L), median (IQR)	125.0 (105.0–148.8)	129.0 (109.0–152.8)	0.448
PLT (10^9^/L), median (IQR)	157.5 (112.0–227.5)	172.0 (111.8–225.3)	0.003
ALT (U/L), median (IQR)	40.0 (16.0–104.5)	23.0 (13.0–42.8)	0.007
AST (U/L), median (IQR)	55.0 (27.0–133.5)	30.0 (17.0–79.5)	0.008
TBIL (μmol/L), median (IQR)	21.1 (14.7–39.1)	11.6 (6.8–22.7)	<0.001
DBIL (μmol/L), median (IQR)	11.8 (5.9–24.9)	4.7 (2.6–10.4)	<0.001
BUN (mmol/L), median (IQR)	8.1 (4.7–13.7)	7.3 (4.2–14.6)	0.602
Cr (μmol/L), median (IQR)	98.0 (71.6–228.6)	80.4 (61.0–213.3)	0.541

**Table 3:** Comparison of prediction results of different model test sets under optimal feature subset.

Performance metrics	Logistic regression	Random forest	BPNN	SVM	Decision tree	XGBoost
Cross-validation accuracy	0.739	0.798	0.732	0.813	0.762	0.791
Test accuracy	0.813	0.871	0.801	0.942	0.871	0.873
AUC	0.831	0.901	0.781	0.871	0.821	0.832
Recall score	0.832	0.971	0.931	0.971	0.951	0.911
Precision score	0.902	0.891	0.851	0.951	0.883	0.896
F1 score	0.861	0.901	0.881	0.931	0.934	0.952

Abbreviations: AUC, area under curve; BPNN, back propagation neural network; SVM, support vector machine; XGBoost, extreme gradient booster.

**Table 4:** The optimal subset of clinical features of the SVM model.

Feature subset no.	Sex	Age	SOFA score	GCS score	CRP	PLT	ALT	TBIL	DBIL	Accuary (%)
1	—	—	√	—	—	—	—	—	—	73.9932
2	—	—	√	—	—	√	—	—	—	82.5591
3	√	√	—	—	—	√	—	—	—	84.9822
4	√	—	√	—	√	—	√	—	—	88.3014
5	—	—	√	√	—	√	√	√	—	89.3364
6	√	—	√	—	√	√	—	√	√	91.0953
7	√	√	√	—	√	√	—	√	√	96.3613
8	√	√	√	√	√	√	—	√	√	94.0921
9	√	√	√	√	√	√	√	√	√	93.3381

## Data Availability

The datasets used and/or analyzed during the current study are available from the corresponding author upon reasonable request.

## Nomenclature

ALI: Acute lung injury
ALT: Alanine transaminase
AMY: Serum amylase
AP: Acute pancreatitis
APACHE II: Acute physiology and chronic health evaluation II
ARDS: Acute respiratory distress syndrome
AST: Aspartate transaminase
BNP: B-type natriuretic peptide
BPNN: Backpropagation neural network
BUN: Blood urea nitrogen
C-index: Concordance index
COVID-19: Coronavirus disease 2019
CRP: C-reactive protein
Cr: Serum creatinine
DBIL: Direct bilirubin
DBP: Diastolic blood pressure
DCA: Decision curve analysis
GCS: Glasgow coma scale
Hb: Hemoglobin
HR: Heart rate
ICU: Intensive care unit
LIP: Serum lipase
MAP: Mean arterial pressure
MODS: Multiple organ dysfunction syndrome
PCT: Procalcitonin
PLT: Platelet
RBC: Red blood cell
ROC: Receiver-operator characteristic
RR: Respiratory rate
SBP: Systolic blood pressure
SOFA: Sequential organ failure score
SVM: Support vector machine
TBIL: Total bilirubin
WBC: White blood cell
XGboost: Extreme gradient booster network.

## Supporting Information

Supporting Information 1 Tripod checklist. Completed TRIPOD checklist for prediction model development and validation.

Supporting Information 2 Table S1. Assessment of multicollinearity among the independent variables, showing acceptable variance inflation factors (VIFs) for all predictors.

Supporting Information 3 Figure S1. Percentage of missing data for each variable prior to imputation. Figure S2. Feature selection using LASSO regression and cross-validation to identify the optimal *λ*-value and determine the most predictive subset of variables.
