# Supplementary material for: Clinical Use of Nomogram Based on Machine Learning for Diagnosis Prediction of Acute Respiratory Distress Syndrome in Patients With Acute Pancreatitis
Source: Mediators Inflamm. 2025 Nov 17;2025:5610316. doi: 10.1155/mi/5610316 (PMC12643695; doi:10.1155/mi/5610316)
Supplement: Supporting Information 2 — Table S1. Assessment of multicollinearity among the independent variables, showing acceptable variance inflation factors (VIFs) for all predictors. [file 5610316.f2.docx]

**Additional file 1: Supplementary Table 1**

**Clinical use of nomogram based on machine learning for diagnosis prediction of acute respiratory distress syndrome in patients with acute pancreatitis**

**Hongjie Hu^1,2^, Yuxin Wang^3^, Yaqin Song^1,2^, Shuhui Wu^1,2^, Dayong Li^1,2^, Liang Jing^1,2^, Lei Qin^4^, Zhaohui Xia^4^, Wei Zhu^1,2*^**

1. Department of Emergency Medicine, Tongji Hospital, Tongji Medical College, Huazhong University of Science and Technology, Wuhan, Hubei, PR China;

2. Department of Intensive Care Medicine, Tongji Hospital, Tongji Medical College, Huazhong University of Science and Technology, Wuhan, Hubei, PR China;

3. School of Biomedical Engineering, Guangzhou Medical University, Guangzhou, Guangdong, PR China;

4. School of Mechanical Science & Engineering, Huazhong University of Science and Technology, Wuhan, Hubei, PR China.

*** Correspondence:**

Wei Zhu, MD, PhD. Department of Emergency Medicine, Department of Intensive Care Medicine, Tongji Hospital, Tongji Medical College, Huazhong University of Science and Technology, 1095 Jiefang Ave, Wuhan, Hubei, PR China, 430030 (Phone: 8627.6363.9013; [tjjzkzw512@163.com](mailto:tjjzkzw512@163.com))

Supplementary Table 1: Assessment of multicollinearity among the independent variables.

| Variable | VIF | SQRT VIF | Tolerance | Squared |
| --- | --- | --- | --- | --- |
| Sex | 1.076 | 1.037 | 0.930 | 0.0706 |
| Age, year | 1.108 | 1.053 | 0.9903 | 0.0975 |
| SOFA score | 1.464 | 1.210 | 0.683 | 0.3169 |
| CRP, mg/L | 1.030 | 1.015 | 0.971 | 0.0291 |
| PLT, 10^9^/L | 1.313 | 1.146 | 0.762 | 0.2384 |
| TBIL, μmol/L | 5.894 | 2.428 | 0.170 | 0.8303 |
| DBIL, μmol/L | 5.933 | 2.436 | 0.169 | 0.8315 |
| Mean VIF | 2.545 |  |  |  |
