# Supplementary material for: Clinical Use of Nomogram Based on Machine Learning for Diagnosis Prediction of Acute Respiratory Distress Syndrome in Patients With Acute Pancreatitis
Source: Mediators Inflamm. 2025 Nov 17;2025:5610316. doi: 10.1155/mi/5610316 (PMC12643695; doi:10.1155/mi/5610316)
Supplement: Supporting Information 3 — Figure S1. Percentage of missing data for each variable prior to imputation. Figure S2. Feature selection using LASSO regression and cross-validation to identify the optimal λ-value and determine the most predictive subset of variables. [file 5610316.f3.docx]

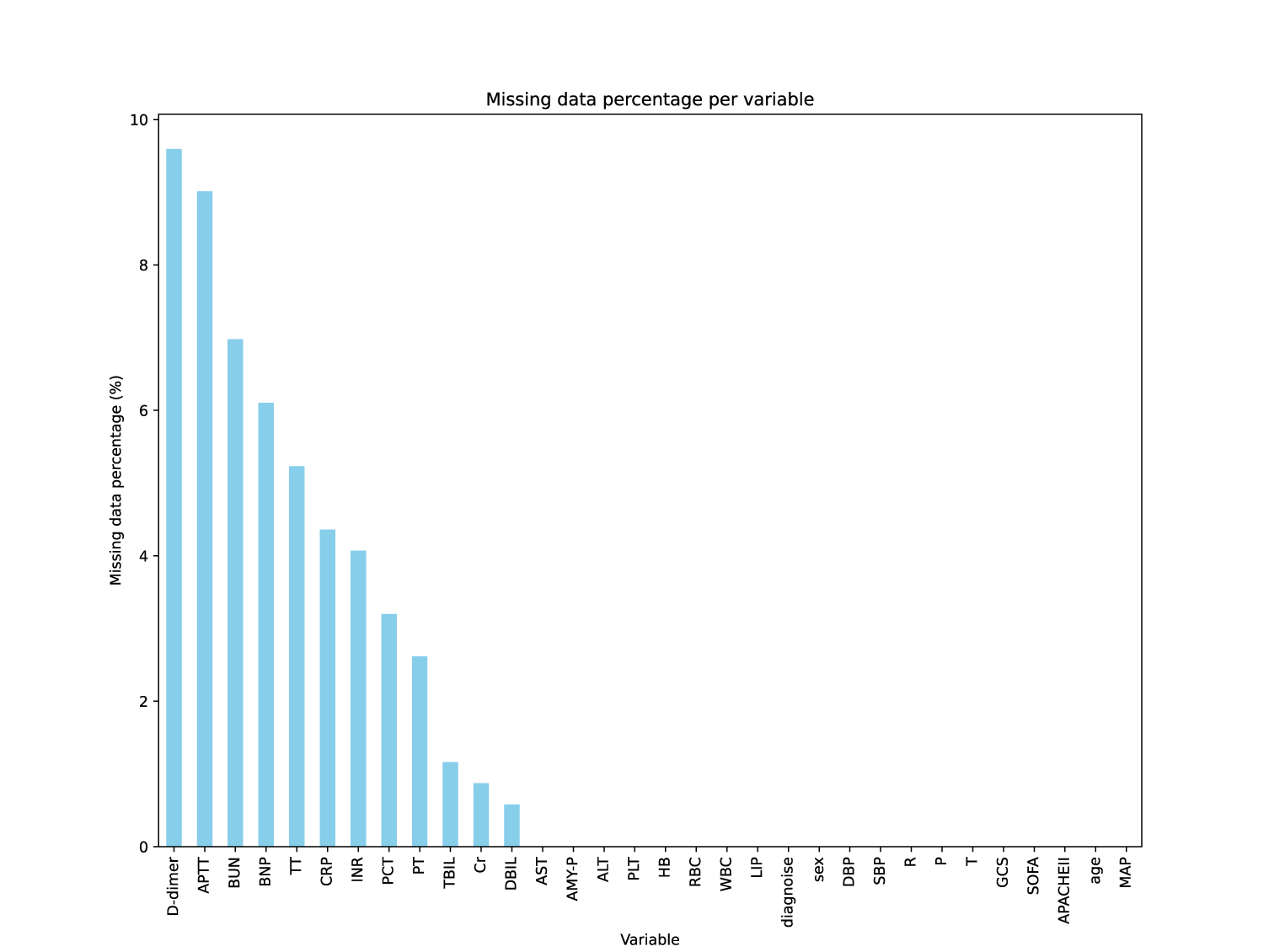


Fig S1 Missing data percentage per variable


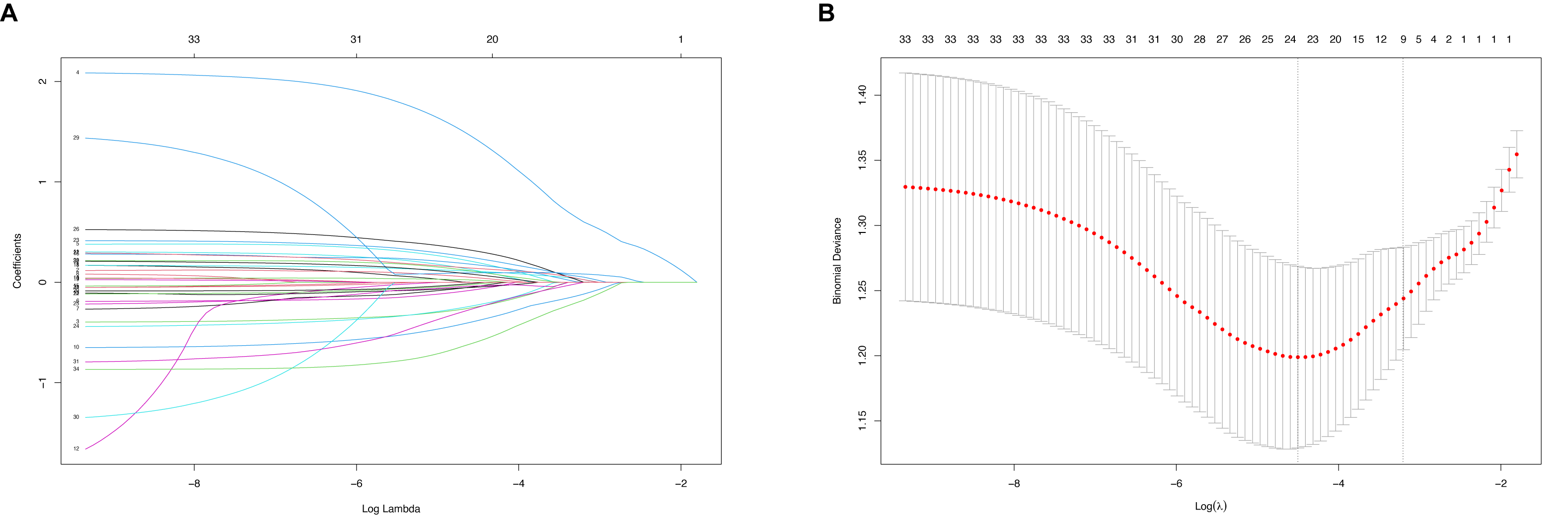


Fig S2 A.The process by which the coefficients of all variables change to 0 in lasso regression; B. Cross-validation of optimal adjustment of λ-value (Left dashed line: minimum mean squared error; Right dashed line: standard error).
